# Cytomegalovirus infection in HIV-infected patients in the era of combination antiretroviral therapy

**DOI:** 10.1186/s12879-019-4643-6

**Published:** 2019-12-04

**Authors:** R. Perello, A. Vergara, E. Monclus, S. Jimenez, M. Montero, N. Saubi, A. Moreno, Y. Eto, A. Inciarte, J. Mallolas, E. Martínez, M. A. Marcos

**Affiliations:** 1 Servicio de Urgencias, Hospital Clínic, Barcelona, Spain; 2 Servicio de Microbiología, CDB, Hospital Clínic, Barcelona, Spain; 3 Servicio de Enfermedades Infecciosas, Hospital Clínic, Barcelona, Spain

## Abstract

**Background:**

Cytomegalovirus infection dramatically decreased with the introduction of antiretroviral therapy. Whether incidence, clinical characteristics and prognosis of cytomegalovirus in HIV infected patients, has changed over time is. scarcely known.

**Methods:**

Retrospective single-center study. Patients included in this study were all HIV infected patients that went to our center for any disease, and were diagnosed with cytomegalovirus, during the period 2004–2015. epidemiological, clinical and laboratory patients variables were collected in a clinical database. Clinical characteristics, incidence of cytomegalovirus and predictors of mortality during the study were assessed. Results were considered statistically significant when *p* < 0.05. All statistical analyses were calculated by SPSS version 20.0 (Chicago, IL,USA).

**Results:**

Fifty-six cases of cytomegalovirus infection, in HIV infected patients were identified during the study period (incidence rate-1.7 cases per 1000 persons/year). The most frequent presentation was systemic illness in 43% of cases. Of note,no patients presented with ophthalmic manifestations. The 30-days mortality was 18%. Predictors of mortality were, in the univariate analysis, admission to the intensive care unit OR 32.4 (3.65–287.06) *p* = 0.0001, and mechanic ventilation 84 OR (8.27–853.12) p = 0.0001, and ART OR 4.1 (0.97–17.31) *p* = 0.044. These variables were assessed by multivariate analysis, and only mechanical ventilation was statistically significant (*p* < 0.05)

**Conclusion:**

Incidence of cytomegalovirus infection was higher than described in the antiretroviral therapy era. Clinical presentation has changed. Mechanic ventilation predicted mortality.

## Background

Cytomegalovirus infection (CMVI), is an AIDS-defining condition that is now much less frequent than in the past due to the widespread availability of combination antiretroviral therapy (ART) [1]. Prior to the introduction of ART approximately 40% of HIV infected patients with advanced immunosuppression suffered from manifestations of an CMVI during their life-time [2].

Different studies suggest that Cytomegalovirus (CMV) is a cofactor for rapid HIV-1 disease progression [3], and CMV has been associated with inflammation and immune activation in HIV-infected patients [4], but detecting CMVI is not an AIDS-defining condition per se. Positive viremia has been reported as a predictor of end-organs CMV disease, and along with low CD4 counts it is associated with increased mortality regardless of the plasma HIV RNA [5]. Among HIV/AIDS patients, CMV may present with a wide range of clinical manifestations and results in a significant exacerbation of morbidity and mortality [6]. The most common manifestations classically reported included retinitis, followed by esophagitis and colitis [7, 8]. It usually developed in patients with very low CD4 cell counts (< 50/mm^3^), and clinicians had to be aware of the possibility of multiorgan involvement [9].

Methods to detect and quantify CMV in clinical samples have been improving over the past few years and have been standardized into clinical routine as important diagnostic tools in patients at risk, including HIV-infected patients [10, 11]. We aimed to review how CMVI may have changed in HIV-infected patients in recent years, now that CMV DNA testing is available for clinical routine. The objectives of this study were to determine the incidence of CMVI in an institution caring for a large cohort of HIV-infected adults, to describe the clinical characteristics of CMVI, and to identify factors associated with a worse prognosis, defined by 30-day mortality and the need for intensive care unit (ICU) admission.

## Methods

### Patients

This is a retrospective single-center study at a large university hospital caring for HIV-infected patients in Barcelona, Spain. All HIV infected patients, with at least one visit in the HIV clinical database, presenting with any symptoms which required hospital admission, and resulted in the diagnosis of CMVI (1st episode), were included in this study. If some patients had to be transferred to another hospital or left the hospital without medical consent, they were excluded from the study. The follow-up time ended when the patient was discharged. We chose the period starting on 1 January 2004 because of the availability of CMV DNA testing from that time onwards. Censorship was 31 December 2015 for the patients not diagnosed with CMV unless death or loss of follow-up.

### Definitions

CMVI is defined as virus isolation or detection of viral proteins (antigens) or nucleic acid in any body or fluid or tissue [13]. CMV disease consists of “end-organ disease” and CMV syndrome. To define “proven CMV end-organ disease”, the presence of appropriate clinical symptoms and/or signs are required together with documentation of CMV in tissue from the relevant organ by histopathology, virus isolation, rapid culture, immunohistochemistry, or DNA hybridization. There was no checking for CMV infection in the absence of symptoms. Definitive diagnosis of tissue-invasive disease relies on detection of CMV in the tissue specimens, however; biopsy obtention is not always feasible (the diagnosis of tissue-invasive CMV disease should be confirmed by immunohistochemistry or in situ DNA hybridization. However, this information was not available in the patients included in our study). CMV syndrome is defined as the presence of fever (> 38 °C) for at least 2 days in a 4-day period associated with the presence of leucopenia, thrombocytopenia or an increase in transaminases, plus evidence of CMV replication [12, 13]. As per clinical routine protocol, all HIV-infected patients diagnosed with CMV were checked for fundoscopy to rule out retinal CMV involvement.

### Clinical variables

For the purpose of this study, the following epidemiological, clinical and laboratory variables were collected: sex, age, associated co-morbidities [14], route of HIV transmission, smoking, alcohol, use of illicit drugs, treatment with ART, previous opportunistic infections, most recent CD4 and CD8 lymphocytes, most recent plasma HIV viral load, co-infection with hepatitis C virus (HCV), target organ of CMV, the presence of fever, need for mechanical ventilation, the total number of white blood cells, platelets, hemoglobin, liver profile, plasma CMV viral load at admission to ICU and 30-day mortality.

### Laboratory studies

Molecular detection of CMV was performed by real-time quantitative PCR ELITechGroup NANOGEN, Italy) after extraction of DNA with DSP Virus/Pathogen Midi kit (Quiagen, Germany) in a QIAsymphony automated platform (Qiagen, Germany. The PCR was done on plasma, BAL fluid and biopsy tissue. The BAL samples were inoculated into human fibroblasts (MRC5) and incubated for 4 weeks on a roller drum at 35 °C. CMV was identified on the basis of cytopathic effect in cell cultures, and their identification was confirmed by staining with fluorescein-conjugated monoclonal antibodies as was previously described (Monofluo Kit CMV,BioRad, France) [15]. Bacterial identification and antibiotic susceptibility tests, and viral culture, were performed according to EUCAST recommendations [16]. *Pneumocystis jirovecii* was detected by Gomori methenamine silver staining, we did not use PCR. Some patients presented insolation in BAL *P jirovencii* and CMV, but we could not affirm that it was affectation of the end organ since it was not possible to perform pulmonary biopsy for CMV. The detection of Toxoplasma gondii was done by serology.

### End point

We aimed to determine the incidence of CMVI in the ART era, the global incidence, divide it into the periods of 2004–2010 and 2011–2015, describe the clinical characteristics of CMV, and identify factors associated with a worse prognosis as defined by 30-day mortality and the need for ICU admission.

### Statistical analysis

Categorical variables were expressed as frequencies and percentages, and continuous variables as median and interquartile range (IQR). For independent samples, Student’s t-test or U-Mann Whitney test as appropriate was used to evaluate the relationship between quantitative variables. The chi-squared test was selected to evaluate the relationship between qualitative variables. To calculate the incidence rate we used the number of new cases of CMVI during the specified time interval, in the numerator, divided by the summed person-years of observation during the time interval, in the denominator.

Factors associated with ICU admission and mortality were assessed by multivariate analysis (including some analytic and demographic baseline characteristics as covariates). Results were considered statistically significant with *p* < 0.05. All statistical analyses were calculated by SPSS version 20.0 (Chicago, IL, USA).

## Results

### Incidence

Out of 7155 adults cared for between 1 January 2004 and 31 December 2015 contributing to 32,198 HIV patient-years of follow-up care, 56 cases of CMVI were identified in the study period. The incidence rate was 1.7 cases per 1000 patient-years, with a median follow up of 4.5 IQR(2.7–6.3) years The incidence rate of CMVI increased significantly from the 2004–2010 period, 0.6 cases per 1000 patient-years, (14 cases in 4293 patients, contributing to 22,865 patient-years) to the 2010–2015 period, 4.5 cases per 1000 patient-years, (42 cases in 2862 patients contributing to 9333 patient-years), (*p* < 0.001).

### Patients

The median age was 39 (IQR 15.8) years old, 44 cases (79%) were males, 6 (11%) were co-infected with hepatitis C virus (HCV) (Table 1). The median CD4 was 47.0 (IQR 212) cells/μl, the median viral load (VL) of HIV was 228,350 (IQR 689725) copies/ml. The median CMV VL in blood was 40,829 (IQR 85) copies/ml, and the mean CMV VL in BAL was 58.7 (IQR 1466.7) copies/ml. CMVI was diagnosed within 3 months after being diagnosed with HIV in 20 (36%) patients, 36 patients were known to be more chronically (defined as more than 3 months apart) HIV-infected prior to CMVI. Twenty six patients were not on antiretroviral therapy at the time of the diagnosis of CMVI. Eighteen were antiretroviral-naive, while eight had previously taken antiretroviral therapy. For those patients on antiretroviral therapy (*n* = 30), 25 were on a protease inhibitor-containing regimen, 4 on a non-nucleoside reverse transcriptase inhibitor-containing regimen, and 1 on an integrase inhibitor-containing regimen. Of the 30 patients on antiretroviral therapy when CMVI was diagnosed median (IQR) cummulative exposure to antiretroviral therapy was 40 (9–100) months. Among these 30 patients on antiretroviral therapy when CMVI was diagnosed, 20 (66%) of them had previously interrupted antiretroviral therapy for longer than 3 months.

**Table 1:** Characteristics of the patients according system involvement of the whole cohort *n* = 56

Variable	Overall n = 56°	Respiratory involvement *n* = 17	Systemic involvement *n* = 24	Digestive involvement *n* = 8	Neurological *N* = 4
Age in years, Median (IQR)*	39 (31–47)	39 (31–47)	38 (31–50)	35 (28–42)	36 (34–51)
Sex, male n, (%)	44 (79)	13 (78)	21 (87)	7 (88)	1 (25)
On HAART**, yes n, (%)	14 (25)	1 (6)	7 (29)	5 (63)	4 (100)
Fever n, (%)	33 (59)	12 (71)	18 (75)	1 (13)	2 (50)
ICU admission n, (%)	19 (34)	8 (47)	7 (30)	1 (13)	2 (50)
Mechanical Ventilation, yes n, (%)	12 (21)	6 (35%)	3 (13)	1 (13)	1 (25)
Mortality n, (%)	10 (18%)	3 (18%)	4 (17)	1 (13)	1 (25)
Cd4 cells/mm3, Median (IQR)*	47 (18–85)	19 (10–45)	63 (22–97)	67 (43–206)	23 (14–69)
Cd8 cells/mm3, Median (IQR)*	514 (272–828)	356 (189–860)	514 (289–653)	727 (494–941)	319 (123–649)
HIV viral load cop/ml × 10^5^ Median, (IQR)*	2.84 (0.5–6.8)	1.27 (0.49–4.03)	3.1 (0.8–9.1)	6.6 (0.95–18.4)	1.21 (0.1–4.8)
CMV*** blood VL Median (IQR)*copies/ml × 10^3^	3.7 (1.09–24.9)	3.9 (1.2–43.6)	1.2 (0.7–13)	1.2 (0.56–31.1)	0.4 (0.1–122)
LDH° IU/L values [< 240], Median (IQR)*	657 (463–1000)	891 (597–1333)	650 (427–809)	578 (441–865)	430 (351–454)

*Interquartile range. ** Highly active retroviral activity ***Cytomegalovirus °Lactate dehydrogenate. °Three patients were not included (1 cardiac, dermatologic and hematological involvement)

Sixteen (28%) patients had previously presented with an opportunistic infection, the most frequent being *P. jirovecii* in seven cases. A total of 38 (68%) patients reported consumption of tobacco smoking, alcohol, illicit drugs, or a combination of the three. Twenty (36%) patients had some co-morbidity associated with their HIV infection, the most common one being chronic obstructive pulmonary disease in 8 (14%) cases. Nineteen (34%) patients were admitted to the ICU and 14 of them required mechanical ventilation (Table 1).

### Clinical presentation

The most frequent clinical presentation were systemic symptoms with fever, cough and general malaise, in 43% of the cases, This was followed by respiratory infection in 30% (mainly pneumonia) and gastrointestinal in 14% (colitis and esophagitis) (Table 1). The most common clinical symptom was fever (59%). No patient had acute retinitis or other ophthalmic involvement. The first choice of treatment for CMVI was ganciclovir in 49 patients (88%), valganciclovir in four and foscarnet in three. Patients under ART treatment had the best improvement.

CMV was found in the blood of 38 (68%) patients, 14 (30%) BAL, and 4 (7%) in intestinal tissue. The diagnosis of CMV was obtained by PCR from blood exclusively in 38 (68%) patients, PCR from BAL exclusively in 5 (9%), isolation of the virus from BAL culture exclusively in 5 (9%), and PCR from intestinal biopsy in 4 (7%). In 4 patients, both the PCR and the culture from BAL were positive for CMV.

The 17 patients who developed respiratory symptoms were diagnosed with CMVI, PCR from blood, and the patients with colitis symptoms, by PCR from the biopsy of the affected organ. CMV was the only pathogen recovered in 13% of all the cases. The most frequent microorganism found together with CMV was *P. jirovecii* (37%) followed by *T. gondii* (14.%). *P jirovecii* was identified in 21 patients, but not all of them had clinical presentation at admission, some of them had systemic presentation as fever, general malaise, etc.

The CMV end-organ disease could only be detected in 4 patients, in which the virus was found in tissue biopsy.

### Predictor factors of mortality

The 30-day mortality was 18% (10 patients) and 50% of them had respiratory-associated infection. One patient died in a conventional room and the rest (9 patients) in the ICU. The mortality of the patients admitted to the ICU was 47%. Drug use, previous opportunistic infection were related with ICU admission (X^2^ = 7.165; *p* = 0.007; X^2^ = 4.464, *p* = 0.035, respectively). The predictive variables associated with mortality, that were identified in the univariate analysis were; ICU admission 32.4 (3.65–287.06) *p* = 0.0001, mechanical ventilation 84 (8.27–853.12) p = 0.0001, and ART 4.1 (0.97–17.31) *p* = 0.044, these variables were assessed by multivariate analysis. Only mechanical ventilation was statistically significant (*p* < 0.05), (Table 2).

**Table 2:** Factors associated with higher mortality at day 30 in patients with CMV infection (n = 56)

Type of analysis	Univariate OR (95% CI)	Multivariate OR (95% CI)
Age in years, older than 39.	0.611 (1.52–245) *p* = 0.485	
ART*, yes	4.1 (0.97–17.31) p = 0.044	
ICU admission, yes	32.4 (3.65–287.06) p = 0.0001	
Mechanical Ventilation, yes	84 (8.27–853.12) *p* = 0.0001	84 (8–853) p = 0.0001
HIV viral load, less than 284,000 copies/ml	2.77 (0.638–12.1) *p* = 0.163	
CD4, less than 30 cells/mm^3^	0.56 (0.13–2.52), *p* = 0.411	
CMV*** BAL VL, less than 5300 copies/ml	1.5 (0.85–2.64), *p* = 0.121	
CMV**** blood VL less than 3700 copies/ml	3.8 (0.67–13.11) P 0.114	
Co morbidities, yes	1.25 (0.3–5) *p* = 0.755	

* antiretroviral therapy***Cytomegalovirus bronchoalveolar viral load, ****Cytomegalovirus blood viral load

Bold formatting represents significant *P* values

The patients with co-infection by *P. jirovencii* presented no greater mortality than the non-coinfected ones (X^2^ = 0.07; *p* = 0.792).

The relationship between CMV viral load in blood and viral load in BAL and mortality was not statistically significant (t = − 1.73; *p* = 0.09), mortality was (t = − 2.18; *p* = 0.07). Neither the CD4 count nor the value viral load was significant (Table 2). Regarding the patients admitted to the ICU, the value of CMV viral load in blood was not related to mortality (*p* < 0.424) even if they had high viral loads.

## Discussion

Our study shows the incidence of acute CMV in recent years (1.7 cases per 1000 patient-years), which is higher than previously reported in the early years of cART (0.6 cases per 1000 patients-year) [17], but lower than the pre-ART era (7.34 cases per 1000 patients-year) [18]. This incidence increase is a matter of concern as it may reflect an increment of susceptible population, since there are still many patients with late diagnosis of HIV infection, which leads to a delay in the initial treatment with ART. Regarding clinical manifestations, the systemic affectation in the form of bad general condition and fever was the most common, followed by respiratory manifestations, which is highly consistent with previous literature [19–21]. In our study, despite the high number of patients with immunosuppression (CD4 < 200 cells/μl), we have not found any cases of ophthalmic manifestation of CMV. Retinitis has been the most common manifestation of CMVI, accounting for about 85% of all the cases [22], although other authors have also found a estimated 80–90% decline on the incidence of retinitis in the ART era [23, 24], probably because of the earlier initiation of ART at HIV diagnosis [25]. ophthalmoscopic examination and rigorous eye check up should be a compulsory practice in every case of HIV/AIDS patients having CD4 count less than 100cells/μl and in those patients with detectable CMV viremia, even in the absence of any vision-related complaints.

Although patients receiving ART clinically evident end-organ CMV disease may no longer be a common problem thanks to the increasing success of ART, there is growing evidence that the impact of the CMV chronic infection remains highly relevant. This may be explained by the immune activation and systemic inflammation caused by CMV increasing the risk of HIV-related morbidity and mortality [26, 27]. Despite this, our study did not show a significant association between CMV viral load and mortality. In our study we were unable to show a poor outcome predictive value of CMV viral load in blood, or viral load in BAL. To our knowledge, this finding has not been previously reported and our results suggest that further research should be conducted to better understand its clinical relevance.

Given the nonspecific symptoms of CMVI and the high mortality of these patients, it may be necessary to consider requesting a diagnostic test for CMV when any HIV patient consults with a widespread and/or infectious systemic affectation even if it is respiratory symptoms and fever. If the patient presents with respiratory symptoms, BAL must be considered since CMV isolation from BAL samples showed to be a predictor of death [28]. This is because, as is known, CMV may be present in the BAL in a patient affected by *P jirovencii*, although we can not really assert that CMV has a pathogenic value in this coinfection, since it is essential to confirm CMV disease In a biopsy of the affected organ, so always treatment for CMV was started [29]..

We need to know the distinction between CMV end-organ disease and CMV viral syndrome, the major difference is the detection, by biopsy, of CMV vat the tissue.

Our study showed that patients with CMVI, coinfection by *P. jirovencii* did not present greater mortality than the non-coinfected ones. These results should be interpreted with caution, likely because the sample is small.

For patients admitted to the ICU, it should be noted that they had already been diagnosed with CMVI before the admission, where a further increase in the rate of reactivation by CMV among critically ill patients is already known [30, 31]. Our study showed a 30-day mortality of 18% and the main cause was respiratory infection, which contrasts with the study from Lichter et al. [32] where the cardiovascular and neurological events, attributable to the role of CMV coinfection in vascular/organ degenerative disorders in HIV-infected subjects, were the main cause of mortality. However, this study was performed in a different setting in which the patients were ART treated and no evidence of CMVI was present and the follow-up was much longer.

### Limitations

Among the limitations of our study, we acknowledge that this is a single-center, retrospective study, however, our center is a reference center for HIV care around 5000 patients/year. We gathered a relatively high number of patients, followed in a similar way, through several years of follow-up. The tissue biopsy was not always feasible, especially in patients with poor general condition. In addition, diagnosis was based on laboratory confirmation of CMVI. Due to the number of patients we cannot affirm that the mortality was high.

## Conclusion

We found an incidence of CMVI higher than that previously described. CMVI in our cohort predominantly affects undiagnosed or untreated HIV-infected patients, for whom the benefit of combination antiretroviral therapy does not exist. Systemic manifestations were the most common presentation. Neither CMV viral load in BAL or in blood, were identified as risk factors for mortality. These findings may be taken into account to increase early HIV diagnosis and early CMV monitoring.

## Data Availability

The datasets generated and/or analysed during the current study are not publicly available due to the fact that we do not wish to share our dataset since they are part of patients’ medical history. However, they are available from the corresponding author on reasonable request.

## Abbreviations

ART: Antiretroviral therapy
BAL: Bronchoalveolar lavage
CMV: Cytomegalovirus
CMVI: Cytomegalovirus infection
